# Radiological insertion of automated low flow ascitic pump (alfapump^®^) system for management of medically refractory ascites

**DOI:** 10.1259/bjrcr.20170025

**Published:** 2017-06-20

**Authors:** Salil Karkhanis, Robert Jones, Andrew Willis, Eoghan Mccarthy, Zergham Zia, Homoyoon Mehrzad, Joanne O'rourke, Andrew Holt, Dhiraj Tripathi

**Affiliations:** ^1^Department of Interventional Radiology, Queen Elizabeth Hospital, University Hospital Birmingham Trust, Birmingham, UK; ^2^Department of Hepatology and Liver Transplant Unit, Queen Elizabeth Hospital, University Hospital Birmingham Trust, Birmingham, UK

## Abstract

Ascites is well-documented sequelae of liver cirrhosis with significant impact on survival in this group of patients. Among the many established management strategies for the same is the use of an implantable mechanical device, called alfapump^®^ (Sequana Medical, Zurich, Switzerland), that removes ascitic fluid by pumping it from the peritoneal cavity to the urinary bladder. Until recently, this device has been surgically placed under general anaesthesia. We describe successful interventional radiological implantation under conscious sedation in three patients with minimal complications. This device can serve as an alternative to transjugular intrahepatic portosystemic shunt for the management of refractory ascites; however, further studies are required to understand the device better.

Ascites is a well-documented sequelae in patients with liver cirrhosis occurring in approximately 60% of patients within 10 years of diagnosis.^1^ This can predispose to subacute bacterial peritonitis, hepatorenal syndrome and poor prognosis with 50% mortality at 3 years.^2^ Apart from managing aforementioned complications, management principles include salt and fluid restriction with diuretics, paracentesis, transjugular intrahepatic portosystemic shunt (TIPSS) and more definitively orthotopic liver transplantation (OLT).^3^ Failure of medical therapy in approximately 10% of patients leads to the diagnosis of refractory ascites, and consideration of other treatment modalities.^4^ Large volume paracentesis (LVP) would be the next management strategy of choice; however, LVPs can result in post paracentesis circulatory dysfunction, due to rapid decrease in effective arterial volume. This can cause rapid reaccumulation of ascites, hepatorenal syndrome, dilutional hyponatremia and increased mortality risk.^5^ Frequent LVPs may lead to consideration of a TIPSS procedure to decompress the portal hypertension, which results in secondary increase in sodium and fluid excretion.^6^ TIPSS procedure has its own set of complications, most importantly, 25–30% incidence of hepatic encephalopathy (HE).^7^

The alfapump^®^ system (Sequana Medical AG, Zurich, Switzerland) is an implantable, mechanical device that provides a means for continuous and more definitive ascitic drainage than LVP. It also serves to avoid complications associated with a TIPSS procedure, such as, HE. Until now, this device has been implanted in the operating room under general anaesthesia. We report three cases of alfapump systems placed in the interventional radiology suite under conscious sedation and local anaesthesia.

## The device and procedure

**Figure 1.**
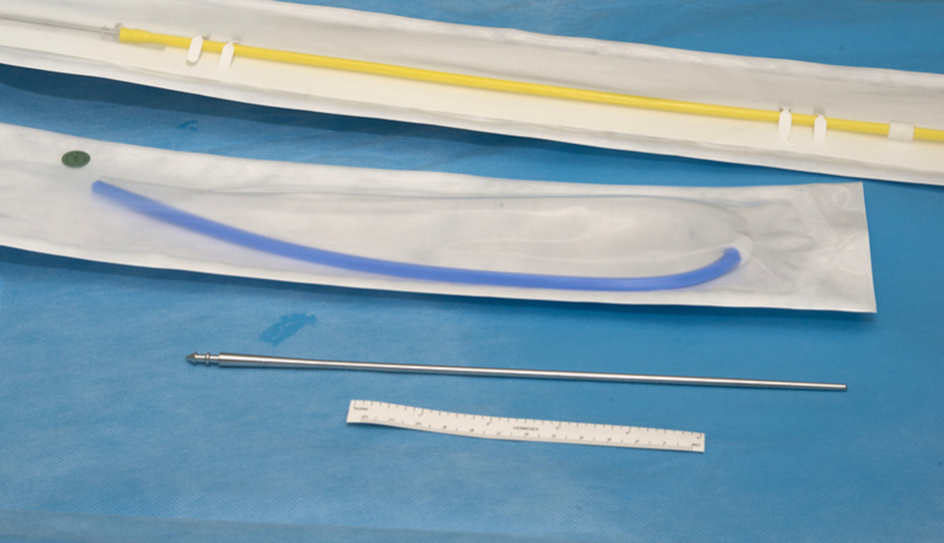
16F blue (peritoneal) and yellow (urinary bladder) catheters along with a metallic tunneling device.

**Figure 2.**
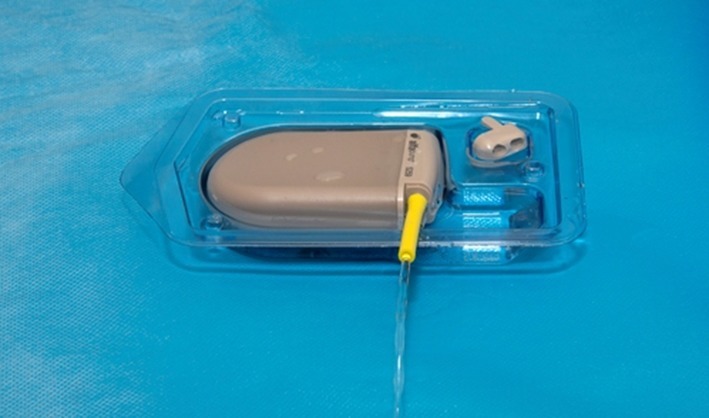
Priming the control unit of the alfapump^®^. The device is submerged in sterile saline and turned on remotely.

The alfapump system drains the ascitic fluid from peritoneal cavity into the urinary bladder, which can then be expelled during micturition. The system consists of two 16F silicone catheters, one a multi-sideholed catheter situated in the peritoneal cavity and the other a soft pigtail catheter positioned in the urinary bladder. Both catheters have Dacron cuffs to allow the tubes to embed within the soft tissues of the anterior abdominal wall following the creation of a subcutaneous tunnel, similar to tunnelled vascular access catheters. Both catheters are connected to a subcutaneous mechanical pump unit running off rechargeable radiofrequency coils, which are recharged daily by the patient (Figures 1 and 2). It is charged by placing a hand-held device over the radiofrequency coil which is located in the control panel. The patient will receive notification (by text message) if the device has not been charged. Nominated team members receive weekly emails detailing volumes of ascites removed. The target volumes can be adjusted in clinic.

Prior to the procedure, patients were reviewed in both hepatology and interventional radiology clinics where they were briefed on the device and consented for the procedure. Patients were admitted to the hepatology ward the day prior to the procedure. A urinary catheter was placed under aseptic technique and clamped 3 hours prior to the procedure to ensure bladder distension. Diuretics were discontinued for at least 7  days and 500 mg of oral ciprofloxacin was given for antibiotic prophylaxis, on the morning of the procedure.

**Figure 3.**
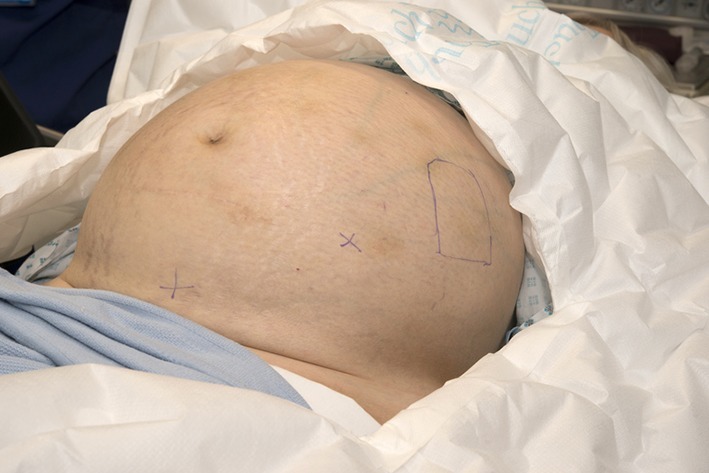
Prior to prepping and draping the positions for the urinary bladder and peritoneal catheter incision sites are marked with crosses. The site of the subcutaneous pocket in the left lower quadrant is also marked.

**Figure 4.**
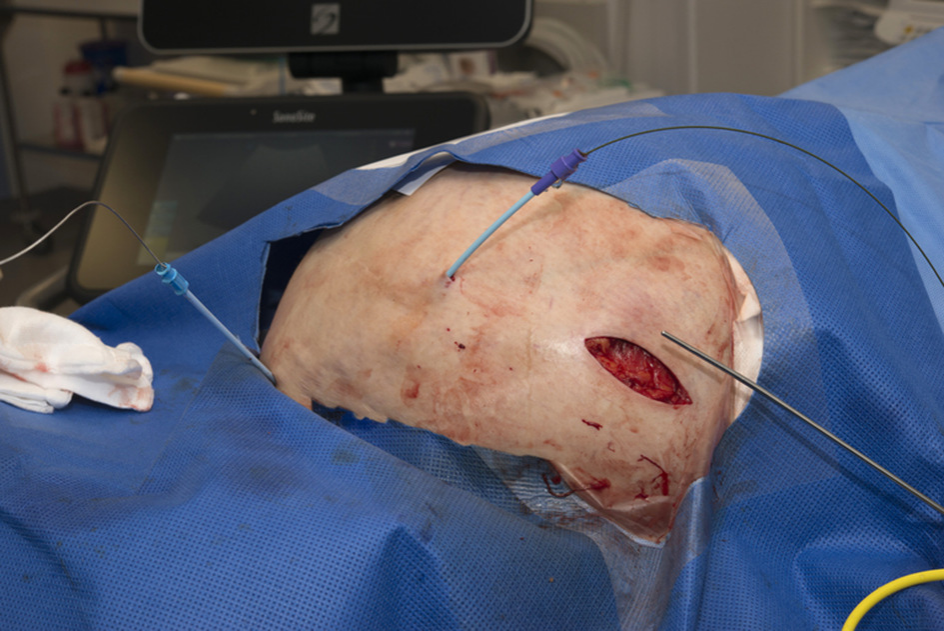
Dilators and guide wires in the urinary bladder and peritoneum following needle access under ultrasound. The metallic dilator overlies the subcutaneous pocket for the control unit.

**Figure 5.**
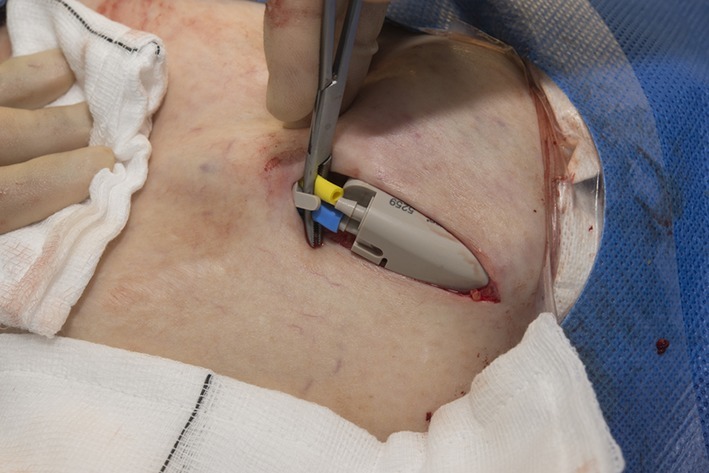
Both the peritoneal (blue) and bladder (yellow) catheters are connected to the control unit. The hook of the locking device which securely attaches the catheters to the control unit is seen adjacent to the forceps.

Patients were positioned supine on the angiography table. An ultrasound examination was performed to document bladder distension and ascites. An appropriate pocket position for the control unit was marked on the skin with a surgical marker, ensuring that it was far enough away from the hip to prevent discomfort in the seated position. Orientation of the control unit was also planned to ensure there would be no kinking of the catheter tubing (Figure 3). If patients were on the active transplant waiting list, the devices were placed in the left side to avoid the possible transplant surgical incision, as per request of our local transplant surgical team. Access points to the bladder and peritoneum were marked to allow for a sufficient length of tunnel. Routine cardiac and respiratory monitoring was initiated. Conscious sedation and analgesia (intravenous midazolam and morphine sulphate) was titrated on the basis of patient requirement. The patients were prepped and draped around the planned skin markings. Local anaesthetic (1% Lidocaine) was given to the skin of the anterior abdominal wall. A 4-cm skin incision was made and the fat layer hydrodissected with a tumescent mixture of saline and 1% Lidocaine. Surgical forceps were used to bluntly create a pocket to snugly fit the control unit. The pocket was packed with sterile surgical gauze (with radio-opaque stripe) to allow haemostasis. Local anaesthetic was then given to peritoneal and bladder access sites. Small incisions were made at these sites and under ultrasound guidance, 18G needles placed into the urinary bladder and peritoneal cavity respectively. Using a modified Seldinger technique access sites were gradually dilated to 18F (Figure 4) over a wire after which peel away introducers were placed. The peritoneal and urinary catheters were then introduced through the peel away sheath. Subcutaneous tunnelling of both catheters was performed under local anaesthetic, allowing both catheters to enter the soft tissue pocket. The catheters were cut to size. A locking device was placed onto the tubing, and the tubing was connected to the control unit, which was then locked (Figure 5). Two 2-0 VICRYL^®^ (Ethicon US, LLC) stay sutures were placed to attach the control unit to the subcutaneous tissue. The fat layers and skin were closed with appropriate absorbable and non-absorbable suture material. Patients were kept in overnight and reviewed the following day by IR and hepatology teams. Patients were maintained on prophylactic oral antibiotic (Ciprofloxacin) for 1 week.

## Results

### Case 1

A 63-year-old female patient was referred to the IR clinic with medically refractory ascites due to Child-Pugh Class B alcoholic-liver disease cirrhosis. The patients’ pre-procedure parameters were as shown in Table 1. The patient did not wish to be considered for OLT or TIPSS, and decision was made to insert an alfapump system.

**Table 1.** Pre-procedural data including indications, patient comorbidities and blood results.

	Case 1	Case 2	Case 3
Aetiology	Alcoholic liver disease	Alcoholic liver disease	Alcoholic liver disease
Child-Pugh score	B	B	C
MELD score	9	10	15
Significant comorbidity	Nil	Nil	Osteoarthritis
Frequency of LVP	Every 4 weeks	Every 3 weeks	Every 4 weeks
Indication for alfapump^®^	Refractory ascites	Refractory ascites, bridge to transplant	Refractory ascites, bridge to transplant
Hb (g l^–1^)	12.1	9.3	10.5
WBC (X 10^9^ l^–1^)	8.1	6.1	5.4
Platelet count (X 10^9^ l^–1^)	185	164	185
INR	1.5	1.5	1.5
Sodium (mmol l^–1^)	130	134	134
Urea (mmol l^–1^)	10.2	7.9	7.6
Creatinine (μmol l^–1^)	58	59	129
GFR	90	90	37
Bilirubin (μmol l^–1^)	16	17	14
ALT (U l^–1^)	31	16	23
AST (U l^–1^)	25	24	30
ALP (U l^–1^)	146	83	134
Albumin (g l^–1^)	43	30	32

The procedure was tolerated well by patient with no significant discomfort. Oozing of ascitic fluid from the peritoneal catheter access site was noted on day 1, which resolved spontaneously after 2 days and was presumed secondary to the elevated abdominal pressure due to volume of ascites.

On day 15, post-implantation patient’s sodium had improved from 130 mmol l^–1^ to 135 mmol l^–1^ and her albumin had decreased minimally from 43 g l^–1^ to 38 g** l^–1^** but remained within the normal range (Table 2). The patient did not require albumin infusion and her nutrition was maintained on oral diet. At last check, patient had 100 litres of ascites drained with a mean daily volume of 74.4 mls in the last 7 days. The patient did not develop renal failure (Table 2).

**Table 2.** Post-procedural parameters including blood results, duration of stay and post-procedure complications

	Case 1	Case 2	Case 3
Hb (g/dl)	11.6	10.2	11.2
WBC (X 10^9^ l^–1^)	6.1	6.2	8.0
Platelet count (X 10^9^ l^–1^)	226	176	241
INR	1.0	1.3	1.4
Sodium (mmol l^–1^)	134	137	133
Urea (mmol l^–1^)	4.5	7.6	13.2
Creatinine (μmol l^–1^)	48	60	158
GFR	90	90	29
Bilirubin (μmol l^–1^)	14	8	19
ALT (U l^–1^)	17	20	23
AST (U l^–1^)	29	33	47
ALP (U l^–1^)	164	86	142
Albumin (g l^–1^)	38	32	43
Duration pump in situ (days)	224	112	289
Number of admission post pump	0	0	4

As the patient’s overall nutrition improved significantly following drainage of ascites, combined with decreasing pump volume, the decision was made to switch off the pump 224 days post implantation. The pump was electively explanted on the patient’s wishes immediately thereafter.

### Case 2

A female patient aged 54 years, with Child-Pugh Class B cirrhosis secondary to excess alcohol intake, was referred to IR clinic with medically refractory ascites. Pre-procedure parameters were as documented in Table 1. The patient had previous encephalopathy, hence was not a candidate for TIPSS. Following much discussion, she was placed on the active liver transplant waiting list and decision was made to insert the alfapump system as a bridge-to-transplant.

The procedure was tolerated well by the patient with no significant discomfort.

The patient suffered with leakage of ascites from the peritoneal incision, initially 1 week after the procedure and then twice more during follow up. This was managed by increasing the pump output and by aspirating the subcutaneous fluid pocket to dryness. Intermittently the patient complained of bladder spasms (likely due to “dry pumping”), which resolved after adjusting the pump settings.

The patient also suffered with one episode of cellulitis near the skin incision, which responded to oral antibiotic therapy.

There were no significant adverse biochemical outcomes (Table 2). The patient underwent OLT 112  days after alfapump implantation. During this time, 77.7 litres of ascites was drained with a mean of 84.6 ml in the final 7 days up to transplant. On day 14 after implantation patient’s sodium levels had improved from 134 mmol l^–1^ to 137 mmol l^–1^ and her albumin levels had improved slightly from 30 g l^–1^ to 32 l^–1^. Patient required only 500 ml of 20% albumin infusion over the course of 112 days to maintain her nutritional status.

The patient underwent successful OLT and the pump was explanted during the same surgical episode.

### Case 3

A 54-year-old female patient, with Child-Pugh C cirrhosis secondary to alcoholic liver disease, was referred to IR clinic for medically refractory ascites.

Pre-procedure parameters were as outlined in Table 1.

The patient tolerated the procedure well, with no complaints of discomfort.

The patient had two episodes of cellulitis (day 32 and 64) and one episode of urinary tract infection (month 8), which required antibiotics and hospital admission.

The patient had small volume ascitic fluid leakage through the pump wound at day 30 and moderate to large volume leakage, again after 4 months, with a large subcutaneous fluid pocket forming around the pump. This was attributed to a migrated bladder tube (seen on ultrasound); hence, pump revision was carried out on day 120 and both bladder and peritoneal tubings were changed.

Bloods on day 15 demonstrated an improvement in albumin; however a persistent acute kidney injury was noted (on background of chronic renal impairment), most likely consequent to hepatorenal syndrome (Table 2).

The patient continued to have persistent ascites, in spite of what seemed like adequate pump volume (1100 ml/day), and required 3 LVPs in a 6-month period. Due to patient choice, the pump was finally explanted 289 days after implantation. The patient died 315 days later due to sequelae of background liver pathology.

## Discussion

Ascites that is refractory to medical management is typically treated by LVP or in selected patients portosystemic shunts, most commonly TIPSS. While TIPSS is more effective at removing ascites compared with paracentesis, it results in greater risk of HE, and overall morbidity and mortality due to its invasive nature.^8^

The need for a less invasive, yet more continuous and definitive method of ascitic drainage led initially to the development of the peritoneo-venous Denver Shunt, and more recently the alfapump.

The Denver peritoneo-venous shunt that has been used to pump ascites from the abdomen through a one-way valve into the systemic venous system.^9^ This system does not reduce the circulating volume but merely redistributes it to the intravascular space; hence pre-existing end stage renal failure is one of the contra-indications.

The introduction of the alfapump has led to a controlled, permanent method of draining ascitic fluid, compared with the redistribution by Denver Shunt.

The efficacy of this device has been looked at in multiple studies. One randomized controlled trial involving 15 patients (alfapump* vs* paracentesis),^10^ and 3 prospective multi-centre studies,^11–13^ demonstrated a significant reduction in average number of LVPs per month and the average volume drained in each LVP. This was noted in two out of three patients in our series. The third patient continued to require LVPs due to pump dysfunction. All the studies noted a transient acute kidney injury, which resolved spontaneously or with conservative management and a non-significant reduction in serum albumin levels. This was seen in one of our patients as well.

The main complication to consider is peritonitis, which could be spontaneous, as with all cirrhosis patients or related to the pump itself and may ultimately require explantation of the device. In the safety study by Bellot et al, 13 (32.5%) alfapump had to be explanted, 7 for difficult to control infections and 1 for wound dehiscence. Eight patients died during the study, three due to sepsis.^11^ Case 3 in our series experienced intermittent skin cellulitis and an episode of urinary tract infection, which were partly the reasons for eventual explantation of device.

Another complication is that of ascitic fluid leakage which was documented in up to 7.5% of cases in the same study.^11^ In most cases this is self-resolving, as seen in all cases in our series. However, following our experience with slightly prolonged ascitic fluid leak in cases 2 and 3, we now drain the abdomen to an estimated residual of 1–2 litres at time of placement. This allows safe placement of the peritoneal catheter within fluid while minimizing the pressure on the peritoneal lining. The same study reported problems with peritoneal or bladder catheters in up to 22.5% of cases and pump failures in 5% of cases.^11^ Case 3 in our series experienced this complication, which required reverting to LVP, to drain any further ascites.

In 2 of our 3 cases, the alfapump served its purpose satisfactorily. In one case, it was used as a bridge to OLT, and in the second case, it led to a significantly improved fluid balance and nutrition in the patient, and the pump was eventually switched off and electively explanted.

To date all reported cases have been carried out surgically in the operating room under general anaesthesia. The skill set and experience of the interventional radiologist lends itself perfectly for implantation of the alfapump device. Broken down to its simplest components, the procedure consists of two Seldinger punctures, the fashioning of two soft tissue tunnels and the creation of a subcutaneous pocket, which is similar but slightly larger than would be required for a Port-a-cath placement. The combination of conscious intravenous sedation and analgesia with local anaesthesia infiltration, was found to be adequate for implantation of this device thus avoiding general anaesthesia and need for an anaesthetist. The procedure was tolerated very well under these conditions in our small series.

Small trials have demonstrated physiological and quality of life improvements with early placement of the Denver shunt.^14,15^ It is possible that when the patient selection and drainage parameters are perfected, similar results may be seen with the alfapump device.

## Conclusions

In our early experience, insertion of alfapump is well tolerated under local anaesthesia and conscious sedation, and carried out entirely by minimally invasive technique by IR, without the need for surgery or general anaesthesia.

We believe, the skills set in IR are apt for successful placement of this device and the results and complications noted in our series are within the spectrum published in literature.

## Consent

Ethical approval for this study was given by the Institutional Review Board and Novel Therapeutics Committee. Informed consent was also obtained from all individual participants included in the study.
